# *In vivo* visualization of burn depth in skin tissue of rats using hemoglobin parameters estimated by diffuse reflectance spectral imaging

**DOI:** 10.1117/1.JBO.29.2.026003

**Published:** 2024-02-15

**Authors:** Md. Anowar Parvez, Kazuhiro Yashiro, Yuki Nagahama, Yasuyuki Tsunoi, Daizoh Saitoh, Shunichi Sato, Izumi Nishidate

**Affiliations:** a Tokyo University of Agriculture and Technology, Graduate School of Bio-Applications and Systems Engineering, Tokyo, Japan; b Tokyo University of Agriculture and Technology, Department of Biomedical Engineering, Tokyo, Japan; c National Defense Medical College Research Institute, Division of Bioinformation and Therapeutic Systems, Saitama, Japan; d Kokushikan University, Graduate School of Emergency Medical System, Tokyo, Japan

**Keywords:** tissue oxygen saturation, methemoglobin saturation, total hemoglobin, diffuse reflectance spectral imaging, canonical discriminant analysis, burn depth

## Abstract

**Significance:**

Burn injuries represent a global public health problem that kills an estimated 180,000 people annually. Non-fatal burns result in prolonged hospitalization, disfigurement, and disability. The most common, convenient, and widely used method for assessing burn depth is physical or visual examination, but the accuracy of this method is reportedly poor (60% to 75%). Rapid, correct assessment of burn depth is very important for the optimal management and treatment of burn patients. New methods of burn depth assessment that are inexpensive, simple, rapid, non-contact, and non-invasive are therefore needed.

**Aim:**

The aim of this study was to propose an approach to visualize the spatial distribution of burn depth using hemoglobin parameters estimated from spectral diffuse reflectance imaging and to demonstrate the feasibility of the proposed approach for differentiating burn depth in a rat model of scald burn injury.

**Approach:**

The new approach to creating a spatial map of burn depth was based on canonical discriminant analysis (CDA) of total hemoglobin concentration, tissue oxygen saturation, and methemoglobin saturation as estimated from spectral diffuse reflectance images. Burns of three different degrees of severity were created in rat dorsal skin by 10-s exposure to water maintained at 70°C, 78°C, and 98°C, respectively. Spectral images for dorsal regions were acquired under anesthesia immediately after burn injury and at 24 h, 48 h, and 72 h after injury.

**Results:**

Most areas of images in the group with skin exposed to 70°C water and 98°C water were classified as 70°C burn and 98°C burn, respectively. In contrast, no significant difference between areas classified as 78°C burn and 98°C burn from 24 h to 72 h was evident in the group with skin exposed to 78°C water, suggesting that burn depth was heterogeneous.

**Conclusions:**

The proposed approach combining diffuse reflectance spectral imaging and CDA appears promising for differentiating 70°C burns from 78°C burns and 98°C burns, and 98°C burns from 70°C burns and 78°C burns at 24 to 72 h after burn injury in a rat model of scald burn injury.

## 1 Introduction

Burns are one of the most serious, dangerous, and unpredictable forms of trauma. In the management and treatment of burns patients, assessment of burn depth is crucial. Clinicians typically classify burns according to depth as superficial burns, superficial dermal burns (SDBs), deep dermal burns (DDBs), and deep burns (DBs).^1^ Superficial burns affect only the superficial layer of the epidermis^2^ and so are typically painful, but they heal without scarring and require no special care.^3^ SDBs are also associated with the epidermal layer, but are more painful, involving blister formation, weeping, and scar tissue. SDBs are very susceptible to infection and therefore require special treatment and care to prevent infection, but do not require surgery.^3^^,^^4^ DDBs, also known as deep partial-thickness burns, involve the epidermis and a portion of the dermis, extending 2 to 3 mm into the deep dermis.^4^ While less painful, DDBs form more scar tissue. Special care is needed, and surgery may be required.^3^ DBs, also known as full-thickness burns, involve all layers of the epidermis and dermis, resulting in little to no pain due to massive nerve damage. Large areas of skin grafting and special care are required.^3^

Although proper and rapid assessment of burn depth is very important for optimal management and treatment of the burn patient, distinguishing between SDBs, DDBs, and DBs is very difficult because burn depth increases with progression of the burn^5^ and decreases with natural healing. Overestimation of burn depth can lead to unnecessary surgery, while underestimation leads to delayed surgery and prolonged hospitalization.^6^ Early, rapid, correct, and accurate differentiation between SDBs, DDBs, and DBs thus remains a challenge and a top research priority in the field of burns treatment.^7^^–^^10^ Several methods and techniques are used to estimate burn depth. The most common is visual examination by the physician along with a report of pain sensation by the patient, together known as physical examination.^11^ However, the accuracy of this approach is very low, at ∼60% to 75% based on clinical experience.^12^

Histopathological examination allows reliable assessment of burn depth but is invasive.^13^ Other advanced methods for objectively assessing burn depth have been investigated, including indocyanine green dye fluorescence imaging,^14^ infrared thermal imaging,^15^ ultrasound imaging,^16^^–^^18^ laser Doppler imaging,^19^ confocal laser scanning microscopy,^20^ photoacoustic imaging,^21^ spatial frequency domain imaging,^22^ and nuclear magnetic resonance imaging.^23^

Diffuse reflectance spectral imaging (DRSI) is a promising, non-invasive clinical diagnostic technique achieved using simple optical components and devices. DRSI can simultaneously quantify the *in vivo* concentration and oxygen saturation of hemoglobin at each pixel in an image, allowing the assessment of various physiological conditions in living tissues.^5^^,^^6^^,^^24^^–^^29^ Burns can change the skin hemodynamics, showing significant increases in the methemoglobin content within a tissue as well as oxygenated hemoglobin and deoxygenated hemoglobin, depending on the severity and depth of the burn.^30^^,^^31^

We have already developed a DRSI method for quantifying melanin, oxygenated hemoglobin, deoxygenated hemoglobin, and methemoglobin in skin tissues and demonstrated spatiotemporal changes in peripheral hemodynamics in rats during methemoglobinemia.^32^ We also found that canonical discriminant analysis (CDA) with total hemoglobin concentration ($C_{\mathrm{HbT}}$), tissue oxygen saturation (${\mathrm{StO}}_{2}$), and methemoglobin saturation (StMet) is promising for differentiating between degrees of burns injuries.^33^ In this study, we propose an approach to spatially map burn severity in skin tissue using the CDA for values of $C_{\mathrm{HbT}}$, ${\mathrm{StO}}_{2}$, and StMet estimated from DRSI. This study aimed to demonstrate the feasibility of this method for differentiating between burn depths in a rat model of scald burn injury.

## 2 Materials and Methods

### 2.1 Production of Burn Wounds

All experimental procedures were performed in accordance with the protocols approved by the Animal Care Committee of the Tokyo University of Agriculture and Technology (approval nos. R03–185 and R04–136). Thirty-three male Sprague–Dawley rats (10 to 12 weeks old; body weight, 210 to 280 g; Tokyo Laboratory Animals Science Co., Tokyo, Japan) were used in this study. Rats were divided into 70°C burn, 78°C burn, and 98°C burn groups, with 11 rats in each group. Anesthesia was induced in rats with isoflurane (1%, 2  mL/min) and maintained at a depth such that the rat showed no response to toe pinching. After the induction of anesthesia, the dorsal and head regions were shaved, and a depilatory agent containing thioglycolic acid was applied to both regions. Before producing the burn wound, carprofen (5  mg/kg) was injected intramuscularly for pain relief. 70°C burns, 78°C burns, and 98°C burns were induced in rat dorsal skin by exposing skin comprising ∼20% of the total body surface area (4  cm×10  cm) to water maintained at 70°C, 78°C, and 98°C, respectively, for 10 s using a Walker–Mason template.^34^ This protocol has been established, and the results of histological examinations confirmed that the burn severities of 70°C burn, 78°C burn, and 98°C burn correspond to SDB, DDB, and DB.^35^^,^^36^ Immediately after producing the burns, rats were resuscitated with intraperitoneal injection of saline solution (25  mL/kg). Carprofen (5  mg/kg) was injected intramuscularly twice daily for postoperative pain relief. The wound surface was covered with a moist wound dressing sheet (PA1A; Zuiko Medical Corp., Osaka, Japan) and wrapped in bandages (Daiei Co., Osaka, Japan). Each rat was kept in an independent cage at 50% humidity and a temperature of 24°C with ad libitum access to food and water.

### 2.2 Hyperspectral Image Data Collection

**Fig. 1:**
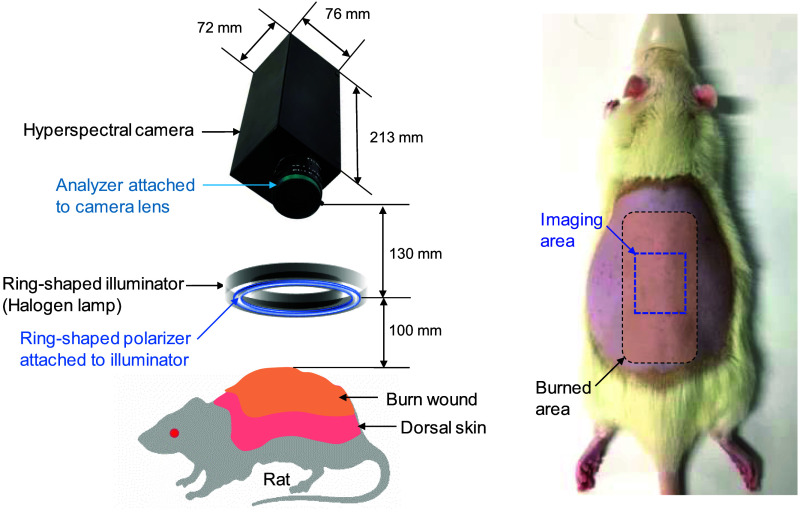
Schematic illustration of the imaging system.

Figure 1 shows the experimental apparatus used in this study. A halogen lamp light source (LA-150SAE; Hayashi Watch Works Co., Tokyo, Japan) was used to illuminate the sample surface via a light guide with a ring-shaped illuminator. Diffusely reflected light was received by a hyperspectral camera (NH-NSD; EBA JAPAN, Tokyo, Japan) with a camera lens to acquire a hyperspectral cube. The camera has an internal optical stage-scanning system comprising a slit, a collimating lens, a transmission diffraction grating, a relay lens, and a two-dimensional (2D) charge-coupled device detector sensor. Hyperspectral image data ranging from 400 to 1000 nm at 10-nm intervals were captured and recorded on a personal computer. The field of view for the system was an area of 32.6×24.5  mm^2^ with 640×480  pixels. Lateral resolution of images was estimated to be 51  μm. A standard white diffuser with 99% reflectance (SRS-99-020; Labsphere, North Sutton, New Hampshire) was used to correct for inter-instrument differences in camera output and the spatial non-uniformity of illumination. A ring-shaped polarizer and an analyzer were set in a crossed Nicols alignment to reduce specular reflection from the skin surface. Total acquisition time for one hyperspectral cube was 13 s. Experiments were performed in a darkroom to prevent the effects of ambient light on spectral imaging. Ambient light therefore had no significant effect on imaging. Sets of spectral images for both dorsal and head regions were acquired pre-burn and immediately post-burn under anesthesia. Subsequent measurements were performed at 24, 48, and 72 h after injury. Sequential spectral images of the dorsal region were acquired from all rats in each group while those of the head region (non-injured skin) were acquired from four rats in each group. Euthanasia was the endpoint of the study and the time of last spectral image data collection. Once sequential data collection had been completed, animals were euthanized by overdose of isoflurane (5%, 2  mL/min) until 1 min after breathing stopped.

### 2.3 Data Processing for Imaging Hemoglobin Parameters

We had already developed an imaging method for quantifying melanin, oxygenated hemoglobin, deoxygenated hemoglobin, and methemoglobin contents in skin tissues and visualizing spatiotemporal changes in peripheral hemodynamics in rats during methemoglobinemia.^33^ This method was leveraged in this study to evaluate concentrations of methemoglobin, oxygenated hemoglobin, and deoxygenated hemoglobin in the rat model of burns injury. Briefly, multiple regression analysis was performed using the absorbance spectrum calculated from the measured diffuse reflectance spectrum of rat skin between 550 and 680 nm as a response variable and the known extinction coefficient spectra of melanin, oxygenated hemoglobin, deoxygenated hemoglobin, and methemoglobin as predictor variables to provide multiple regression coefficients. Concentrations of melanin ($C_{m}$, vol. %), oxygenated hemoglobin ($C_{\mathrm{HbO}}$, vol. %), deoxygenated hemoglobin ($C_{\mathrm{HbR}}$, vol. %), and methemoglobin ($C_{\mathrm{metHb}}$, vol. %) were then determined from regression coefficients using empirical formulas that were deduced numerically in advance. A Monte Carlo simulation (MCS) of light transport^37^ in a human skin model was carried out to numerically establish the empirical formulas. We assumed that whole blood with 150  g/L of hemoglobin represents a 100% volume concentration of total hemoglobin ($C_{\mathrm{HbT}}=100$ vol. %) and is uniformly distributed in the dermis. The volume concentration in this case represents the percentage of blood in a unit volume of dermis. Values of $C_{\mathrm{HbO}}$, $C_{\mathrm{HbR}}$, $C_{\mathrm{metHb}}$, and $C_{m}$ were estimated for each pixel. $C_{\mathrm{HbT}}$ (vol. %), ${\mathrm{StO}}_{2}$ (%), and StMet (%) were also calculated. The sum of the values of $C_{\mathrm{HbO}}$, $C_{\mathrm{HbR}}$, and $C_{\mathrm{metHb}}$ represents the volume concentration of total hemoglobin $C_{\mathrm{HbT}}$. Values of ${\mathrm{StO}}_{2}$ and StMet were calculated as $100\times (C_{\mathrm{HbO}}/C_{\mathrm{HbT}})$ and $100\times (C_{\mathrm{metHb}}/C_{\mathrm{HbT}})$, respectively. All image processing was performed using MATLAB version 2018b (MathWorks, South Natick, Massachusetts).

### 2.4 Statistical Analysis

Only one area was used for each dorsal and head area of each rat for data analysis of hyperspectral images. A region of interest (ROI) of 330×330  pixels was set at the burn wound area in each image, and mean and standard deviation (SD) over the ROI were calculated for analyses of the time courses of $C_{\mathrm{metHb}}\;C_{\mathrm{HbO}}$, $C_{\mathrm{HbR}}$, $C_{\mathrm{HbT}}$, ${\mathrm{StO}}_{2}$, and StMet. Data are thus expressed as mean ± SD. To compare differences in each parameter across burn depth conditions, the mean of three rats in each group was also calculated. An unpaired t-test was performed to compare mean values over samples among groups, with values of P<0.05 considered statistically significant. CDA was performed to discriminate 70°C burns, 78°C burns, and 98°C burns from estimated values of $C_{\mathrm{HbT}}$, ${\mathrm{StO}}_{2}$, and StMet by the proposed method. In this CDA, the time T (s) after burn injury and mean values of $C_{\mathrm{HbT}}$, ${\mathrm{StO}}_{2}$, and StMet over the ROI and their second-order terms were used as predictor variables, while the categories of burn depth (i.e., 70°C burn, 78°C burn, and 98°C burn) were coded as integers and used as response variables. Wilks’ lambda distribution was used to evaluate the significance of separations. The results of discrimination were evaluated by canonical discriminant plots, and the percentage of correct prediction for each sample was evaluated using leave-one-out cross-validation. Discrimination performance was evaluated using a receiver operating characteristic (ROC) curve. Area under the ROC curve (AUC) was calculated to quantify discrimination. In each group, 7 of the 11 rats were used for the training data set to establish the canonical discriminant equations. The remaining four rats were used to provide test data for burn depth classification images.

### 2.5 Visualizing Spatial Distributions of Burn Depths

**Fig. 2:**
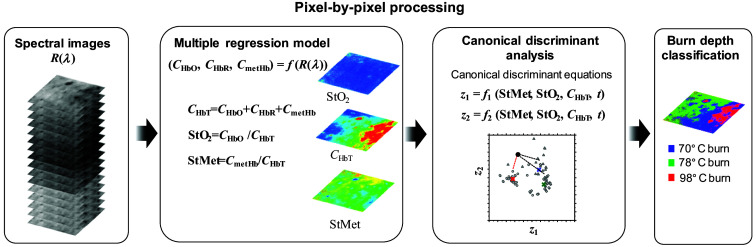
Process of constructing a 2D burn depth classification image.

We proposed a method to visualize the spatial distribution of burn depths based on CDA with images of $C_{\mathrm{HbT}}$, ${\mathrm{StO}}_{2}$, and StMet. Figure 2 shows the process for constructing a 2D burn depth classification image. First, images of $C_{\mathrm{HbT}}$, ${\mathrm{StO}}_{2}$, and StMet are estimated from SDRI of the dorsal skin of a rat using the method based on multiple regression analysis supported by the MCS.^32^ Applying the canonical discriminant equations established by the CDA described in Sec. 2.4 to each pixel of images for $C_{\mathrm{HbT}}$, ${\mathrm{StO}}_{2}$, and StMet, 2D distributions of canonical scores $z_{1}$ and $z_{2}$ are obtained. Pixel-by-pixel calculations of the distance between a coordinate $\left(z_{1},z_{2}\right)$ and the mean value of each burn depth group on the canonical plot finally visualize the spatial map of burn depth.

## 3 Results and Discussion

**Fig. 3:**
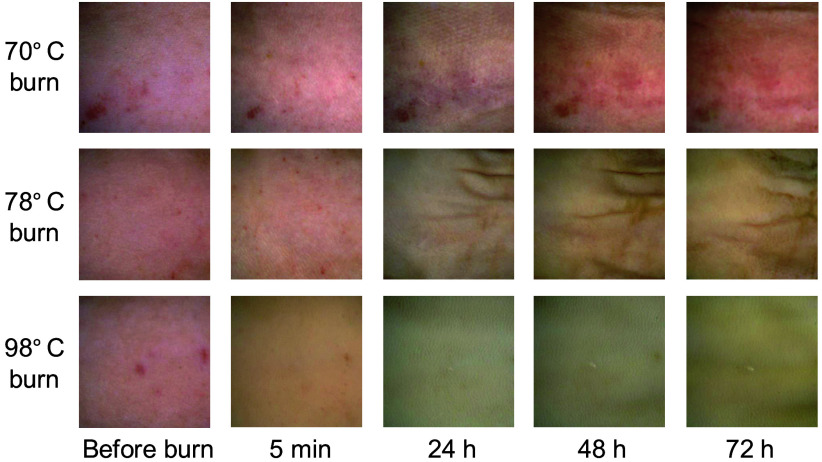
Typical color photographs obtained from rat dorsal skin before burn, and 5 min, 24 h, 48 h, and 72 h after burn injury for 70°C burn, 78°C burn, and 98°C burn.

Figure 3 shows typical color photographs obtained from rat dorsal skin before burn, and 5 min, 24 h, 48 h, and 72 h after burn injury for 70°C burn, 78°C burn, and 98°C burn. The skin color before the burn was a pale pink tone. The skin color for the 70°C burn and the 78°C burn retained a pale pink tone at 5 min after burn injury, but the tone decreased in the skin for the 98°C burn. The skin color for the 70°C burn became a light reddish-purple at 24 h after burn, but the pale pink tone recovered at 48 and 72 h after burn. The skin color for the 78°C burn showed a pale yellowish tone at 24 h after burn, and some wrinkles appeared on the surface which is probably due to shrinkage of the injured skin tissue. The skin color for the 98°C burn became a grayish tone at 24 and 48 h after burn and it changed to slightly yellowish at 72 h.

**Fig. 4:**
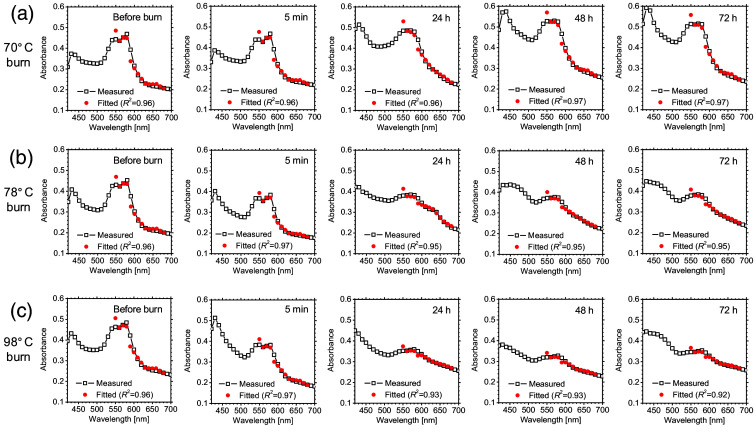
Typical time course of absorbance spectra obtained from rat dorsal skin before burn, and 5 min, 24 h, 48 h, and 72 h after burn injury for (a) 70°C burn, (b) 78°C burn, and (c) 98°C burn.

Figure 4 shows typical time course of absorbance spectra obtained from rat dorsal skin before burn, and 5 min, 24 h, 48 h, and 72 h after burn injury for 70°C burn, 78°C burn, and 98°C burn. Each spectrum is the average value over the ROI in the spectral absorbance image and is compared to the absorbance spectrum fitted by the multiple regression analysis with the molar extinction coefficient spectra of oxygenated hemoglobin, deoxygenated hemoglobin, methemoglobin, and melanin. The absorbance values for 78°C burn and 98°C burn in the range from 610 to 650 nm were slightly increased 24 h after burn injury, which implies increases in $C_{\mathrm{metHb}}$. Each fitted spectrum shows good agreement with the measured spectrum in the 550 to 680 nm wavelength range. The coefficient of determination $R^{2}$, for each fitted spectrum ranged from 0.92 to 0.97, indicating a high goodness of fit. The absorbance values for 78°C burn and 98°C burn in the range from 420 to 540 nm were elevated 48 and 72 h after burn injury, indicating an increase in bilirubin due to hemoglobin breakdown. In this study, the multiple regression analysis was performed using a spectrum between 550 and 680 nm. Therefore, the presence of bilirubin, which has no absorption in this wavelength range, would not affect the goodness of fit.

**Fig. 5:**
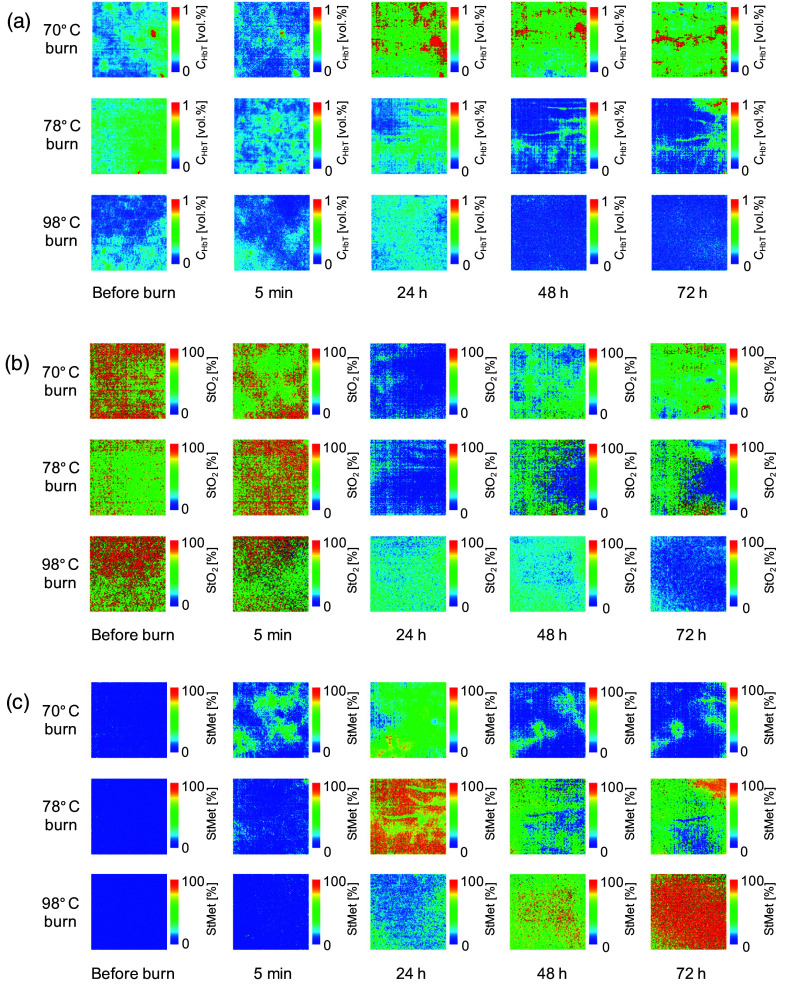
Typical sequential images of (a) $C_{\mathrm{HbT}}$, (b) ${\mathrm{StO}}_{2}$, and (c) StMet before and after 70°C burn, 78°C burn, and 98°C burn in rat dorsal skin.

**Fig. 6:**
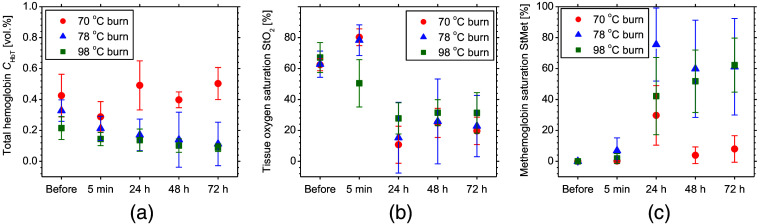
Time courses of (a) $C_{\mathrm{HbT}}$, (b) ${\mathrm{StO}}_{2}$, and (c) StMet before and after 70°C burn, 78°C burn, and 98°C burn in rat dorsal skin averaged over the ROI for each corresponding image obtained from all samples. Plots and error bars show means and SDs of seven rats in each burn injury group (i.e., 70°C burn, 78°C burn, and 98°C burn) in rat dorsal skin.

Figure 5 shows typical sequential images for $C_{\mathrm{HbT}}$, ${\mathrm{StO}}_{2}$, and StMet before and after SDB, DDB, and DB in rat dorsal skin. Figure 6 shows the time courses of $C_{\mathrm{HbT}}$, ${\mathrm{StO}}_{2}$, and StMet before and after 70°C burn, 78°C burn, and 98°C burn in rat dorsal skin averaged over the ROI of each corresponding image obtained from all samples. Plots and error bars show means and SDs for seven rats in each burn injury group (i.e., 70°C burn, 78°C burn, and 98°C burn) in rat dorsal skin. In Figs. 5(a) and 6(a), $C_{\mathrm{HbT}}$ in the 70°C burn group showed different time courses from those in the 78°C burn and 98°C burn groups. In the 70°C burn group, $C_{\mathrm{HbT}}$ increased significantly from 24 to 72 h after burn injury. Conversely, in the 78°C burn and 98°C burn groups, $C_{\mathrm{HbT}}$ remained decreased compared to pre-burn levels from 24 to 72 h after burn injury. $C_{\mathrm{HbT}}$ in 70°C burn has been reported to be significantly increased, peaking at 48 h after burn injury and continuing to increase until at least 72 h.^6^ In contrast, $C_{\mathrm{HbT}}$ in 78°C burn continued to decrease over time. Total hemoglobin has been observed to be elevated in 70°C burn, but stable in 78°C burn.^38^ In thermally injured tissues, microcirculation is progressively disrupted due to tissue destruction, vascular occlusion, microvascular endothelial changes, and thrombus formation. This leads to changes in total hemoglobin concentration in different types of burn injury. One possible explanation for these changes is venous blood pooling due to impaired vascular supply.

In Figs. 5(b) and 6(b), ${\mathrm{StO}}_{2}$ for 70°C burn was increased immediately (i.e., 5 min) after burn injury, then decreased at 24 h, and finally continued to increase at 48 to 72 h to reach its pre-burn level. On the other hand, in the 78°C burn and 98°C burn groups, ${\mathrm{StO}}_{2}$ remained decreased at 24 h after burn injury, then increased slightly to reach the pre-burn level at 48 to 72 h after burn injury. These results are consistent with findings from other researchers for 70°C burn, but the results for 78°C burn and 98°C burn did not match previous findings at 24 h, and finally at 48 to 72 h after burn injury, all types of burn depth showed oxygen saturation at pre-burn levels.^6^ Previous researchers have also reported similar findings to our results that tissue oxygen saturation appeared to be significantly increased immediately after burn, but these differences were not evident at 24, 48, and 72 h after burn.^38^

Figures 5(c) and 6(c) show that StMet for 70°C burn was increased immediately after burn injury and reached the maximum level at 24 h after burn injury, then returned to the pre-burn level by 48 h after burn injury. In contrast, StMet for 78°C burn and 98°C burn started to increase immediately after burn injury and continued to increase at 24, 48, and 72 h after burn injury. The time courses for estimated StMet changed depending on burn depth. The mechanisms underlying increased methemoglobin content in burn wounds have been discussed elsewhere.^39^^–^^41^ StMet appears to be one of the most important parameters for burn depth and provides unique ways to assess and characterize different types of burns, and these findings also suggest that quantifying StMet may provide a critical indicator of the extent of damage within the burn wound.^31^.^39^^,^^42^ Methemoglobin is formed by the oxidation of iron moieties in normal hemoglobin from the ferrous to the ferric state. Levels may thus be increased in injured tissue because of the increased levels of reactive oxygen species (ROS) generated by neutrophils and reactive nitrogen species (RNS), such as nitric oxide.^41^^,^^43^^–^^45^ Temporal differences in the influx of neutrophils into tissues reportedly exist between SDB and DDs in human patients.^30^ Oxidation of deoxygenated hemoglobin and oxygenated hemoglobin by RNS also produces methemoglobin.^46^

**Fig. 7:**
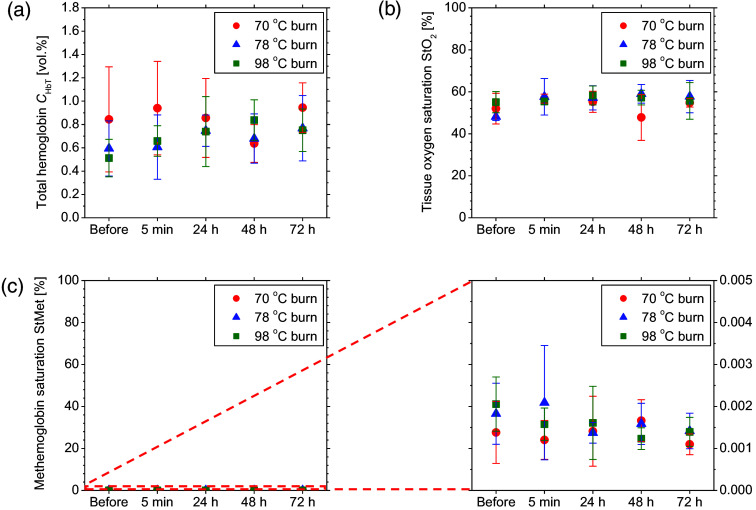
Time courses of (a) $C_{\mathrm{HbT}}$, (b) ${\mathrm{StO}}_{2}$, and (c) StMet for 70°C burn, 78°C burn, and 98°C burn for rat head skin (non-burned site).

Figure 7 shows the time courses of $C_{\mathrm{HbT}}$, ${\mathrm{StO}}_{2}$, and StMet for 70°C burn, 78°C burn, and 98°C burn for rat head skin (non-burned site). In the non-injured head skin distal to the burn wound, there were no significant changes in StMet from pre-burn levels for all (70°C burn, 78°C burn, 98°C burn) groups. An initial decrease in $C_{\mathrm{HbT}}$ was observed in both the 78°C burn and 98°C burn groups, which was followed by gradual recovery for 72 h. On the other hand, $C_{\mathrm{HbT}}$ for the 70°C burn group showed a minimum value at 48 h after burn injury. There was no correlation between changes in $C_{\mathrm{HbT}}$, ${\mathrm{StO}}_{2}$, and StMet observed at non-burn sites and those observed at burn sites. Therefore, the changes in $C_{\mathrm{HbT}}$, ${\mathrm{StO}}_{2}$, and StMet observed at the burn site are specific only to burn depth.

**Fig. 8:**
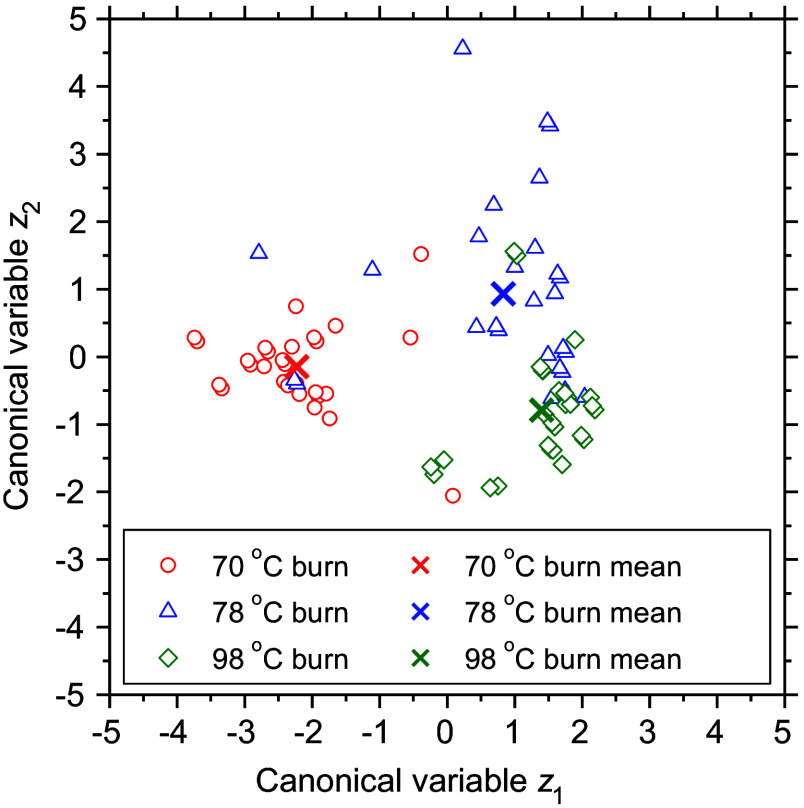
Scatter plots from CDA.

Figure 8 shows the canonical discriminant plots obtained from CDA. The plots showed reasonable separation among 70°C burn, 78°C burn, and 98°C burn over time. In this study, 89.29%, 53.57%, and 92.86% of the original grouped cases for 70°C burn, 78°C burn, and 98°C burn were correctly classified, respectively, compared to 89.29%, 42.86%, and 89.29%, respectively, of cross-validated grouped cases.

**Fig. 9:**
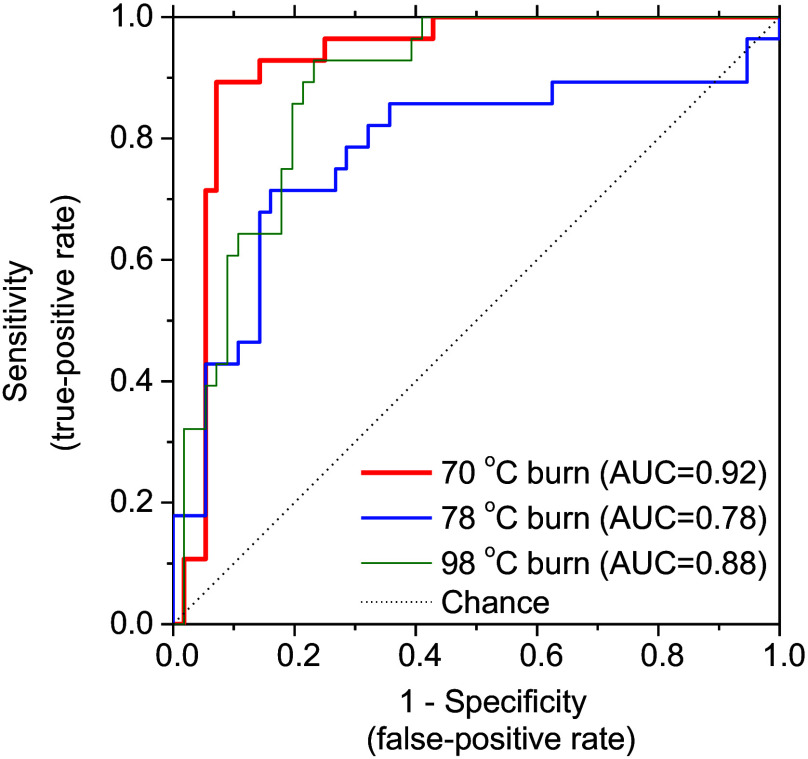
ROC curves of 70°C burn, 78°C burn, and 98°C burn groups.

Figure 9 shows the ROC curves of the 70°C burn, 78°C burn, and 98°C burn groups. AUCs for the 70°C burn, 78°C burn, and 98°C burn groups were 0.92, 0.78, and 0.88, respectively. As expected from the canonical scatterplot results in Fig. 7, ROC curves of the 70°C burn group showed AUCs exceeding 0.9, indicating excellent discrimination accuracy. In the 98°C burn group, the AUC was 0.88, a sign of good discrimination accuracy. On the other hand, AUC for the 78°C burn was <0.8, indicating fair discrimination accuracy. Overall, this discriminatory approach using only hemoglobin derivatives information appears promising for the classification of 70°C burn, 78°C burn, and 98°C burn within 72 h after burn injury.

**Fig. 10:**
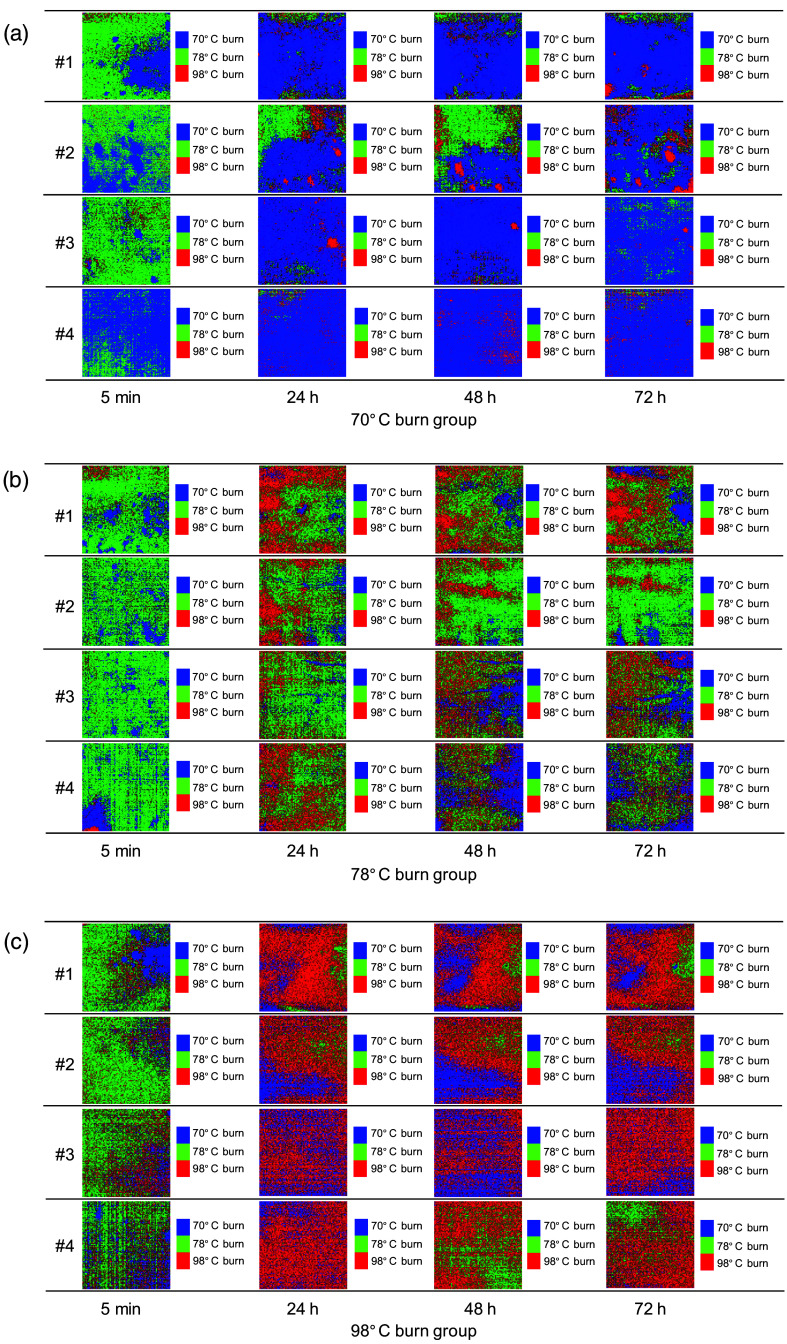
Typical sequential images for burn depth classification obtained from groups of rats with dorsal skin exposed to hot water at (a) 70°C, (b) 78°C, and (c) 98°C.

**Fig. 11:**
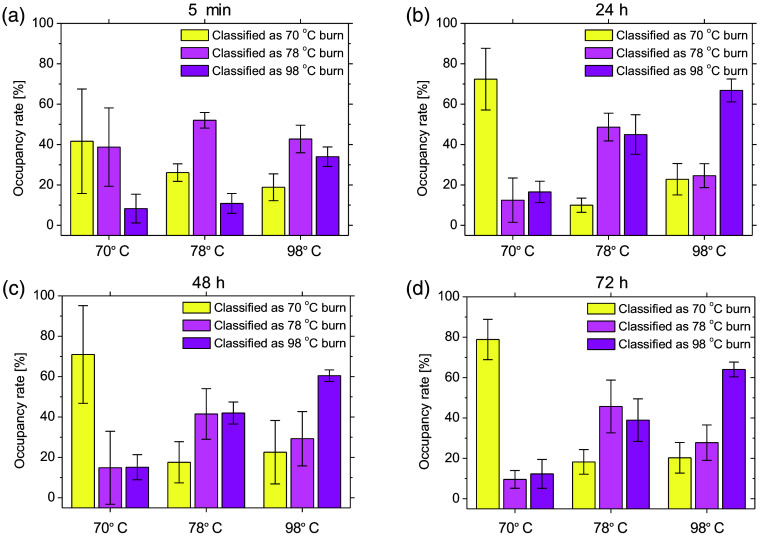
Occupancy rate of pixels classified as 70°C burn, 78°C burn, and 98°C burn in each image averaged over the four samples for each group at (a) 5 min, (b) 24 h, (c) 48 h, and (d) 72 h after burn injury.

Figure 10 shows the typical sequential images of burn depth classifications obtained from the groups exposed to 70°C, 78°C, and 98°C water. Figure 11 shows the occupancy rate of pixels classified as 70°C burn, 78°C burn, and 98°C burn in each image averaged over the four samples for each group at 5 min, 24 h, 48 h, and 72 h after burn injury. For the dorsal skin of rats exposed to 70°C water, the resulting burn depth classification images mostly comprised 70°C burn and 78°C burn regions at 5 min immediately after burn injury. Conversely, areas classified as 70°C burn dominated at 24 to 72 h after burn injury. For the dorsal skin of rats exposed to 78°C water, most areas in burn depth images for all four samples were classified as 78°C burn at 5 min after burn injury, whereas images comprised 70°C burn, 78°C burn, and 98°C burn regions at 24 to 72 h after burn injury. For the dorsal skin of rats exposed to 98°C water, burn depth classification images comprised 70°C burn, 78°C burn, and 98°C burn regions at 5 min after burn injury. Areas classified as 98°C burn dominated at 24 to 72 h after burn injury.

Burn wounds are not usually uniform in depth, and many comprise a mixture of deep and superficial components.^47^^,^^48^ A study using photoacoustic imaging of rat burn wounds induced by the Walker–Mason method showed that the signal representing burn depth was site-dependent, even though uniform heating had been used to create the burn injuries.^35^ This is consistent with the results from burn depth classification images shown in Figs. 9(b) and 10.

Most areas of images from the group with skin exposed to 70°C or 98°C water were classified as 70°C burn or 98°C burn, respectively. In contrast, no significant differences between areas classified as 78°C burn and 98°C burn at 24 to 72 h were seen in the group with skin exposed to 78°C water. One possible explanation is that burn depth changed from 78°C burn to 98°C burn, since burn wounds are dynamic and can progress as well as convert to deeper wounds.^42^^,^^49^

In this study, 2D images of burn depth were created based on the results of CDA with the parameters of hemoglobin derivatives. Investigation with CDA showed reasonable results for discriminating between 70°C burn, 78°C burn, and 98°C burn at 48 to 72 h after burn injury and for differentiating the 78°C burn group from the 70°C burn and 98°C burn groups immediately after burn injury. Similar findings have been reported by other researchers when assessing burn depth using hyperspectral imaging.^49^

In our previous work,^33^ CDA was performed separately at four different time points: 5 min, 24 h, 48 h, and 72 h after injury, and discriminant equations were calculated for each time point. Practically, it would be better to prepare discriminant equations that can be applied to an arbitrary time point. Therefore, in this study, $C_{\mathrm{HbT}}$, ${\mathrm{StO}}_{2}$, and StMet for all time points were combined into one data set for predictor variables of CDA. In addition, elapsed time after the burn injury T was added as a predictor variable. This allows classification of burn severity at any time up to 72 h after injury. The method relies on a small training and validation dataset in this study. Therefore, we performed leave-on-out cross-validation to evaluate the percentage of correct prediction for each sample in CDA with the small dataset. $C_{\mathrm{HbT}}$, ${\mathrm{StO}}_{2}$, and StMet from 21 samples (7 rats × 3 burn severities) were used as the training data set in each CDA in our previous work.^33^ In contrast, this study used $C_{\mathrm{HbT}}$, ${\mathrm{StO}}_{2}$, StMet, and T from 84 samples (7 rats × 3 burn severities × 4 time points) as training data in single CDA. The number of samples for single CDA in this study is larger than that in our previous work.^33^ Therefore, the statistical power of CDA in this study is higher than that in the previous study.

The proposed method cannot yet be claimed to accurately classify 70°C burn, 78°C burn, and 98°C burn based only on the results of this study. We also applied a new approach based on CDA for differentiating burn depth. Burn depth images were obtained from the data set of $C_{\mathrm{HbT}}$, ${\mathrm{StO}}_{2}$, and StMet, allowing 70°C burn and 98°C burn to be easily classified, but showing a mixture of pixels of all burn depths for 78°C burn. We used mean ROI values for $C_{\mathrm{HbT}}$, ${\mathrm{StO}}_{2}$, and StMet as predictive variables to derive the canonical discriminant equations. However, as shown in Fig. 5, $C_{\mathrm{HbT}}$, ${\mathrm{StO}}_{2}$, and StMet displayed spatial heterogeneity. In addition, mean ROIs for $C_{\mathrm{HbT}}$, ${\mathrm{StO}}_{2}$, and StMet could vary between samples. Although the Walker–Mason method is a well-established protocol for inducing burn wounds with relatively high reproducibility, few studies have investigated the spatial uniformity of burn depths induced by this method. We assumed that burn depths in the dorsal skin of rats exposed to 70°C, 78°C, and 98°C water represented SDB, DDB, and DB, respectively, based on the literature.^36^ However, some discrepancies may have existed between assumed and actual burn depths for each sample. Determination of burn depth from histopathological observations of different regions of the burn site at different time points is needed to assign the category names of SDB, DDB, and DB to the response variable in CDA. This should be investigated in future work.

We measured all hemoglobin parameters at 5 min after burn injury because we were interested in the changes in these parameters and the classification of burn depth under the proposed approach based on CDA immediately after burn injury, although differentiating burn depth at 5 min after burn injury offers no real clinical value. The present method lacks depth resolution, since it relies on integrating all the diffuse reflectance information from 550 to 680 nm along the depth direction, which may represent one factor limiting the accuracy of burn depth classifications. Therefore, it is difficult to determine with this method whether the observed chromophore changes originate from the injured tissue alone or from both the injured and underlying non-injured tissues. We simply selected the central area (330×330  pixels) of the measured image as the ROI. Considering the heterogeneity of the estimated images for $C_{\mathrm{HbT}}$, ${\mathrm{StO}}_{2}$, and StMet, it is expected that differences in the size and position of the ROI may affect the model predictions.

When the proposed method is applied to humans, a large number of $C_{\mathrm{HbT}}$, ${\mathrm{StO}}_{2}$, and StMet data for burn sites of different severity should be collected from human patients and used as a training data set for CDA. Because albino rats were used in this study, the amount of melanin was close to 0% at all burn severities. In humans, melanin may be present in superficial burns and SDBs, but in deeper burns, the epidermis may be severely damaged and may slough off. In such cases, there will be no melanin present at the burn wounds.

We also performed spectral imaging of pre-burn dorsal skin and pre- and post-burn head skin, and estimated the values of $C_{\mathrm{HbT}}$, ${\mathrm{StO}}_{2}$, and StMet. The aim of this study was to classify the severity of burn injuries at the burn wound sites. Therefore, we considered three categories in CDA: 70°C burn, 78°C burn, and 98°C burn, but did not include non-burn areas in the data set. Therefore, the burn severity classification map did not include results for non-burned areas. It will be possible to classify the map into four categories of 70°C burns, 78°C burns, 98°C burns, and non-burns by including data sets of $C_{\mathrm{HbT}}$, ${\mathrm{StO}}_{2}$, and StMet obtained from non-burn sites in CDA.

Several researchers have reported methods for near infrared diffuse reflectance analysis of burns.^30^^,^^39^^,^^42^^,^^50^ These methods can detect changes in water content in burn wounds, which would be useful for assessing burn edema. In addition to the absorption properties of burn wounds, scattering properties have also shown utility in the assessment of burn depth.^51^ In this study, empirical formulas for chromophore concentrations were derived from the MCS with a typical spectrum of light-scattering coefficients. Our current method therefore does not account for variations in the light scattering properties of the skin model. However, this spectrum tends to vary from one part of the body to another and may also vary with the age of the individual. Variability in scattering coefficient spectra may thus affect the accuracy of estimating chromophore concentrations. Although this study focused on changes in hemoglobin derivatives, the accuracy of classifying burn depth may be possible using the light-scattering parameter as one variable in CDA.

Early and accurate assessment of the burn depth is important because the current burn management strategy is early excision and skin grafting of all deep dermal and full-thickness burns.^52^ The current standard waiting time for a decision to graft or continue wound care is 3 to 7 days post-burn.^51^ Laser Doppler imaging is the only technique approved by the FDA for burn assessment. It has been shown to reduce surgical workload by eliminating unnecessary surgeries and is used to predict burn healing between 48 h and 5 days post-burn.^12^ The proposed approach with CDA for $C_{\mathrm{HbT}}$, ${\mathrm{StO}}_{2}$, and StMet in burn wounds showed reasonable results for discriminating between 70°C burn, 78°C burn, and 98°C burn at 48 to 72 h after burn injury and for discriminating the 78°C burn group from the 70°C burn and 98°C burn groups immediately after burn injury. Therefore, the proposed method can meet the clinical need for diagnosis as soon as possible after injury and for use in decision making for grafting or continuation of burn wound care.

When using this method to evaluate DBs in human skin, which is thicker than rat skin, it is better to analyze the diffuse reflectance spectrum at longer wavelengths, which has a greater optical penetration depth. The light penetration depth in the range of 550 to 680 nm roughly reflects the sampling depth in this study, but it depends on the absorption coefficient (i.e., the amount of each chromophore) and the scattering coefficient of the tissue in this wavelength range. Therefore, it is difficult to determine with this method whether the observed chromophore changes originate from the injured tissue alone or from both the injured and underlying non-injured tissues. Our results showed that the amount of each chromophore varies depending on the severity of the burn injury. This means that the sampling depth changes depending on the severity of the burn. It has been reported that in deep partial thickness and full thickness burns, the reduced scattering coefficients decrease significantly after burn injury.^51^ The decrease in light scattering will result in an increase in light penetration depth. Several researchers^21^^,^^39^^,^^42^ conducted the experimental study with more granularity of depths than the number of classifiers used in this study. Experiments using a rat thermal injury model with finely segmented burn depths should be conducted in future work.

## 4 Conclusions

In summary, a new method for classifying and imaging burn depth in rats based on SDRI was demonstrated in this study. The proposed approach with CDA for ${\mathrm{StO}}_{2}$, StMet, and $C_{\mathrm{HbT}}$ in burn wounds showed reasonable results for discriminating between 70°C burn, 78°C burn, and 98°C burn at 48 to 72 h after burn injury and for discriminating the 78°C burn group from the 70°C burn and 98°C burn groups immediately after burn injury. The proposed approach combining SDRI and CDA shows potential for differentiating between degrees of burns in a rat model of scald burn injury.

## Data Availability

Data underlying the results presented in this paper are not publicly available at this time but may be obtained from the authors upon reasonable request and through a collaboration agreement.
